# RGS12 represses oral squamous cell carcinoma by driving M1 polarization of tumor-associated macrophages via controlling ciliary MYCBP2/KIF2A signaling

**DOI:** 10.1038/s41368-023-00216-5

**Published:** 2023-02-16

**Authors:** Gongsheng Yuan, Shuting Yang, Shuying Yang

**Affiliations:** 1 Department of Basic and Translational Sciences, School of Dental Medicine, University of Pennsylvania, Philadelphia, USA; 2 The Penn Center for Musculoskeletal Disorders, Perelman School of Medicine, University of Pennsylvania, Philadelphia, USA; 3 Center for Innovation & Precision Dentistry, Penn Dental Medicine and School of Engineering and Applied Sciences, University of Pennsylvania, Philadelphia, USA

**Keywords:** Oral cancer, Tumour-suppressor proteins

## Abstract

Tumor-associated macrophages (TAMs) play crucial roles in tumor progression and immune responses. However, mechanisms of driving TAMs to antitumor function remain unknown. Here, transcriptome profiling analysis of human oral cancer tissues indicated that regulator of G protein signaling 12 (RGS12) regulates pathologic processes and immune-related pathways. Mice with RGS12 knockout in macrophages displayed decreased M1 TAMs in oral cancer tissues, and extensive proliferation and invasion of oral cancer cells. RGS12 increased the M1 macrophages with features of increased ciliated cell number and cilia length. Mechanistically, RGS12 associates with and activates MYC binding protein 2 (MYCBP2) to degrade the cilia protein kinesin family member 2A (KIF2A) in TAMs. Our results demonstrate that RGS12 is an essential oral cancer biomarker and regulator for immunosuppressive TAMs activation.

## Introduction

Head and neck squamous cell carcinomas (HNSCCs) originate from the mucosal epithelium in the oral cavity, which is one of the most common cancers worldwide.^1–4^ Although cancer immunotherapy has been considered a breakthrough, about 50% of patients with tumors cannot have clinical improvement.^5^ Currently, understanding oral cancer is viewed as a complicated tumor microenvironment where numerous immune cells are recruited to form a self-sufficient biological structure.^6–9^

Tumor-associated macrophages (TAMs) are essential components of the tumor microenvironment and the major tumor-infiltrating leukocytes in most cancers.^10,11^ Studies have shown that TAMs involve in cancer-related inflammation, immunosuppression, and immunotherapy.^12–14^ Under different environments, TAMs can be polarized into M1-like or M2-like subtypes.^15,16^ For example, M1 macrophages can be induced by bacterial cellular components such as LPS or IFN-γ to foster inflammation response against tumor cell proliferation and migration. M2 macrophages can be induced by Th2-derived IL-4 and IL-13 to promote tissue repair, angiogenesis, and tumor development.^15^ Importantly, M2-like TAMs are related to unfavorable survival, whereas M1-like TAMs are related to favorable outcomes in patients with oral cancer.^17^ Therefore, understanding the phenotypic and functional changes of M1 and M2 macrophages is crucial for the treatment and prognostic evaluation of patients with oral cancer.

Recently, Singh et al. found that primary cilia are important organelles of bone marrow monocytes and the assembly and length of cilia are altered under different environments, which regulate the phenotype and function of macrophages.^18^ Moreover, primary cilia are critical for tumor progression and microenvironment, and the decreased number of primary cilia is frequently found in oral squamous cell carcinoma (OSCC) tissues.^19,20^ However, what factors control cilia assembly and/or functions in OSCC is unknown.

Interestingly, most recent studies show that regulators of G protein signaling (RGS) proteins can control cilia assembly or functions to regulate signaling transduction and cell activity.^21,22^ For example, RGS5 is located in the cilia and can inhibit sonic hedgehog signaling in stem cells.^21^ RGS18 is highly expressed in the cilia of hair cells, and deletion of RGS18 dramatically reduces cilia numbers.^22^ Forced overexpression of RGS2 enhances ciliary beat frequency in human airway epithelial cells.^23^ However, how RGS proteins regulate cilia and oral cancer pathogenesis remains undefined.

RGS12 belongs to the RGS family, which is highly expressed in macrophages and promotes the M1 macrophage polarization.^24^ Moreover, by analyzing the GenomeRNAi database (http://www.genomernai.org/v17/genedetails/6002), we found that RGS12 is strongly associated with ciliogenesis. RGS12 contains multiple functional domains that may act as scaffolds to activate the target proteins by post-modifications such as phosphorylation, ubiquitination, and SUMOylation.^25–30^ We previously found that RGS12 promotes the phosphorylation and SUMOylation of PTEN to repress cancer cell proliferation and migration.^25^ However, it is unclear whether RGS12 is expressed in TAMs and controls anti-tumor immunity by affecting cilia.

In this study, by investigating the roles of RGS12 on TAMs from mice with oral cancer, we revealed that RGS12 is an essential factor to control immune activation in oral cancer by regulating macrophage polarization and ciliogenesis via MYCBP2/KIF2A.

## Results

### RGS12 is a critical biomarker for the OSCC

**Fig. 1:**
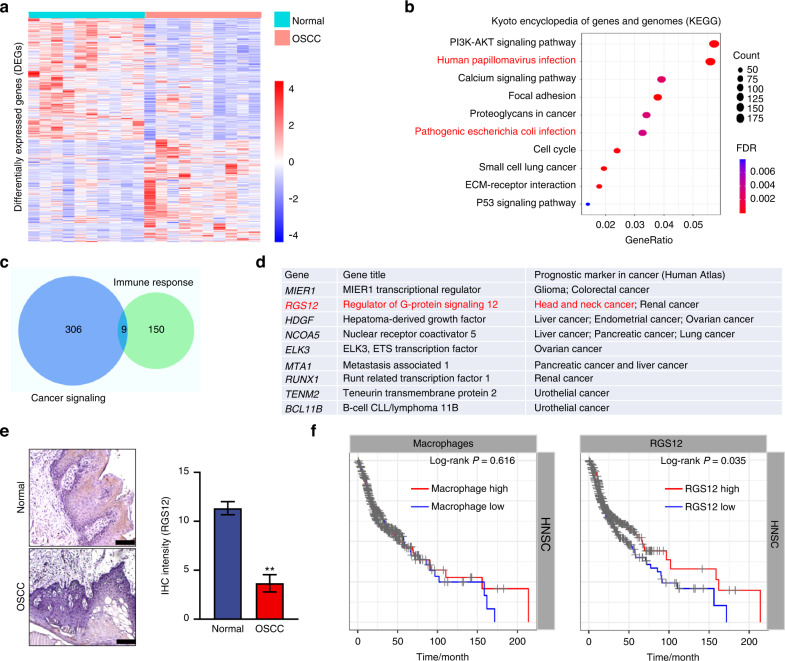
RGS12 is a biomarker of oral squamous cell carcinoma. **a** Hierarchical clustering heat map of DEGs by human normal tissues and OSCC tissues. **b** KEGG pathway enrichment analysis of **a**. **c** Venn diagram of DEGs. Genes in overlapping sets show the differential expression in cancer signaling and immune response. **d** The 9 overlapping genes from **c** were performed by the prognostic markers analyses through the Human Atlas database. **e** Representative images of the immunohistochemical (IHC) staining for RGS12 in the human normal and OSCC tongue tissues. Scale bar, 100 μm. The right panel indicates the quantitative date of relative intensity in **e**. *n* = 3, **P < 0.01. **f** Survival curves of the HNSCC patients with high and low levels of macrophages and RGS12 from the Tumor Immune Estimation Resource (TIMER) platform. Statistical analysis among HNSCC subtypes was performed and compared using the log-rank test and *P* < 0.05 was considered to be significant

To identify the potential biomarkers and repressors for OSCC, we first evaluated the RNA sequencing data in the GEO database (GSE37991). As a result, we identified 9220 DEGs comprising 4 093 upregulated and 5 127 downregulated genes with an FDR of less than 0.05 between normal and OSCC samples (Fig. 1a). Since infection and immunity are closely associated with oral cancer, we then performed the KEGG and GO enrichments of GSE37991 (Supplementary Fig. S1) and found that immune responses such as virus infection and bacterial infection are the key processes during oral cancer, suggesting that the immune microenvironment may be essential for oral cancer development (Fig. 1b). To identify key regulators in the progression of oral cancer, we overlapped the gene enrichments of cancer signaling and immune response from GO enrichments by using the Venn method (Fig. 1c). We identified 9 genes that were overlapped and found that only RGS12 was the specific prognostic marker of head and neck cancer (*P* < 0.001) by analyzing the Human Atlas database (https://www.proteinatlas.org/ENSG00000159788-RGS12/pathology) (Fig. 1d). We then confirmed that the RGS12 expression was significantly downregulated in human oral cancer samples by immunohistochemistry analysis (Fig. 1e). Interestingly, Kaplan-Meier survival analysis indicated that the RGS12 levels in oral cancer were significantly correlated with the survival rate. The patients with more RGS12 expression have higher survival rates (*P* < 0.05). Similarly, the patients with more macrophages in oral cancer tissues exhibited slightly longer survival without statistical significance (*P* > 0.05) in comparison with those with fewer macrophages (Fig. 1f). These findings indicated that RGS12 expression and its immune regulation were significantly correlated with oral cancer. In addition, we found that RGS12 is mainly expressed in macrophages compared to other immune cells (Supplementary Fig. 2). However, we found no difference in RGS12 expression in peripheral cells between the healthy control people and HNSCC patients (Supplementary Fig. 3a). Similarly, we found no difference in RGS12 transcript or protein levels of peripheral blood mononuclear cells between the control and HNSCC mice (Supplementary Fig. 3b). These findings imply that RGS12 expression is altered only in TAMs surrounding oral cancer.

### Deletion of RGS12 in myeloid lineage promotes the progression of oral cancer

**Fig. 2:**
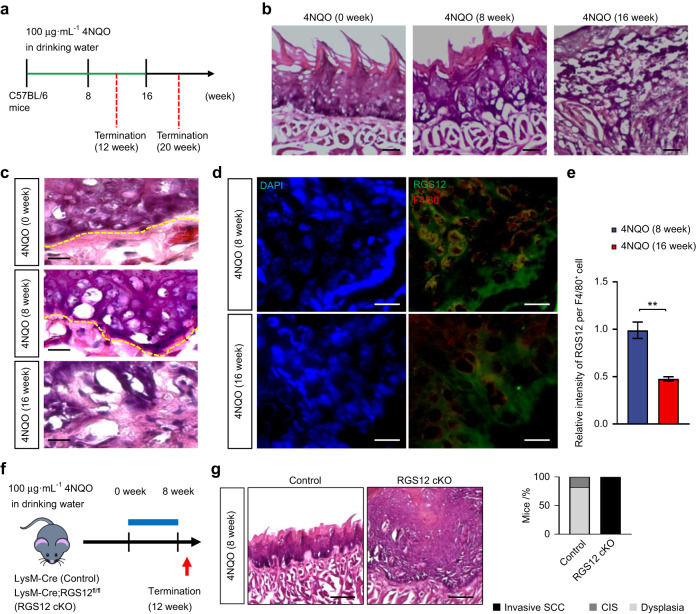
Deletion of RGS12 in myeloid lineage promotes the progression of oral cancer. **a** Diagram of the protocol to generate oral carcinogenesis model. **b** H&E-stained sections of tongue epithelium at 0, 8, and 16 weeks after 4NQO administration. Scale bar, 50 µm. **c** H&E-stained sections of tongue epithelium (around basement membrane, yellow dashed line) at 0, 8, and 16 weeks after 4NQO administration. Scale bar, 20 µm. **d** Immunofluorescence staining for RGS12 and F4/80 expression in 4-week and 8-week 4NQO treated tongue epithelium. Scale bar, 20 µm. **e** The relative intensity of RGS12 per F4/80+ cell as indicated in **d**. **P < 0.01, *n* = 5. **f** Diagram of the protocol for the RGS12 conditional knockout (RGS12 cKO, LysmCre; RGS12^fl/fl^) mice and Control (Ctrl, LysmCre) mice with 8-week 4NQO induction. **g** H&E staining of Ctrl and RGS12 cKO tongue epithelial layers at 8 weeks after 4NQO induction (*n* = 10). Scale bar, 100 µm. The bar graph showed the percentages of the indicated lesion types present in the tongues. SCC, squamous cell carcinoma; CIS, squamous carcinoma in situ

To determine the development of oral cancer in different stages, C57BL/6 mice were given drinking water containing or not containing 4NQO for 8 or 16 weeks and changed to normal drinking water for additional 4 weeks (Fig. 2a). Mice without 4NQO administration showed normal tongue epithelium. However, mice with 8-week 4NQO administration displayed dysplasia, while mice with 16-week 4NQO administration developed invasive squamous cell carcinoma (SCC) in the tongue (Fig. 2b). Moreover, histological examination in higher magnification showed abundant polymorphic epithelial cells with hyperchromatic nuclei, the evidence of dysplasia in 8-week 4NQO treatment. The mice developed invasive SCC after 16-week 4NQO administration with features of cancer cells penetrating beyond the basement membrane (Fig. 2c). To study the functions of RGS12 in the progression of oral cancer, immunofluorescence was performed to determine the RGS12 expression level in macrophages. As expected, RGS12 expression was significantly reduced in F4/80^+^ macrophages from the 16-week 4NQO-induced oral cancer tissues in comparison with that from 8-week 4NQO-induced oral cancer tissues (Fig. 2d, e). To further identify the role of macrophage RGS12 in oral cancer, we used LysmCre;RGS12^fl/fl^ (RGS12 cKO) mice, which were produced by mating LysmCre with RGS12^fl/fl^ mice (Fig. 2f) created previously in our laboratory.^31^ Surprisingly, with 8-week 4NQO induction, histological examination revealed a phenotype of invasive oral cancer in all RGS12 cKO mice compared with the control Cre mice with dysplasia and carcinoma in situ (CIS) (Fig. 2g).

### RGS12 in TAMs drives the polarization of M1 macrophages with increased cilia number and length compared with M2 macrophages

**Fig. 3:**
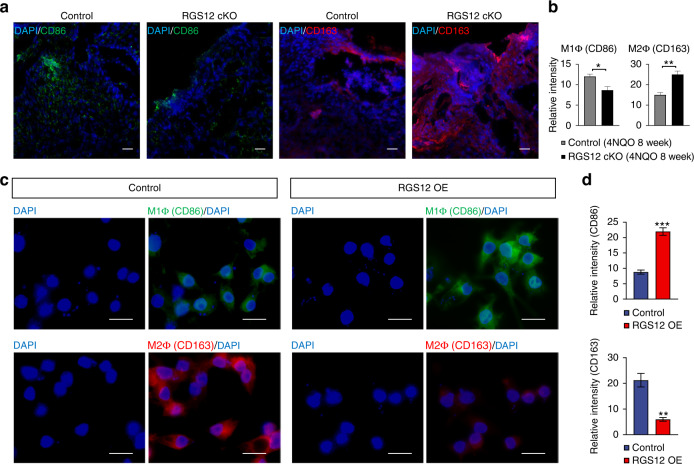
RGS12 in TAMs promotes the polarization of M1 macrophages. **a**, **b** Immunofluorescence for CD86 and CD163 expression to detect M1 and M2 macrophages in tongue epithelial layers from control and RGS12 cKO mice **a**. The right panels **b** indicate the quantitative data of relative intensity of CD86 or CD163 protein. Scale bar, 50 µm. *P < 0.05, **P < 0.01, *n* = 5. **c**, **d** TAMs were obtained from the oral cancer tissues of WT mice. The M1 macrophage (CD86) and M2 macrophage (CD163) were detected by immunofluorescence staining **c**. Scale bar, 20 μm. Statistical data showed the overexpression of RGS12 promotes M1 macrophages but inhibits M2 macrophage polarization **d**. **P < 0.01, ***P < 0.001, *n* = 5

**Fig. 4:**
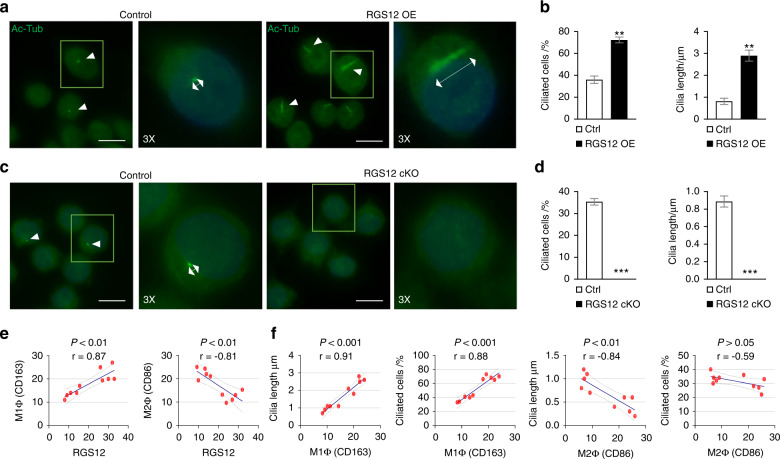
RGS12 in TAMs drives the polarization of M1 macrophages with increased cilia number and length compared with M2 macrophages. **a**, **b** Primary cilia in TAMs transfected with vector control or RGS12 overexpression (OE) vector (48 h) conditions (green, Ac-tubulin for cilia; blue, DAPI for nuclei) **a**. Scale bar, 10 μm. **b** The ciliated cell number and cilia length were calculated from the images in **a**. **P < 0.01, *n* = 5. **c**, **d** Primary cilia in TAMs from the WT control and RGS12 cKO mice **c**. Scale bar, 10 μm. **d** The ciliated cell number and cilia length were calculated from the images in **c**. ***P < 0.01, *n* = 5. **e** Pearson’s correlation analysis indicating RGS12 expression and M1/M2 macrophage markers in TAMs. Pearson’s correlation coefficient (r) and P-values are shown for each analysis (*n* = 5). **f** Correlation between cilia number/length and M1/M2 markers in TAMs. Pearson’s correlation (r) values and *P*-values are indicated within each graph (*n* = 5)

Interestingly, loss of RGS12 inhibits the expression of M1 macrophage (CD86) but promotes the expression of M2 macrophage (CD163) in 8-week 4NQO-induced oral cancer tissues (Fig. 3a, b). To further characterize the function of RGS12 in vitro, we stably overexpressed RGS12 in primary TAMs isolated from oral cancer tissues of wild-type (WT) mice. Consistently, the semi-quantitative analysis of TAMs by immunofluorescence showed significantly increased M1 macrophages (CD86) whereas decreased M2 macrophages (CD163) in RGS12 OE TAMs (Fig. 3c, d). Since RGS12 has a strong relationship with cilia by analyzing the GenomeRNAi database (http://www.genomernai.org/v17/genedetails/6002), we then examined the cilia formation and length in TAMs with RGS12 OE and cKO. Interestingly, the results indicated that RGS12 OE enhanced the ciliated cell number and cilia length (Fig. 4a, b) whereas RGS12 cKO inhibited the ciliated cell number and cilia length in TAMs (Fig. 4c, d). Moreover, by Pearson’s correlation analysis of the fluorescence intensity of RGS12, CD163, and CD86, we found the RGS12 expression is positively correlated with the expression of M1 macrophage (CD86) but negatively correlated with the M2 macrophage (CD163) (Fig. 4e), suggesting that RGS12 OE mainly drives the polarization towards to M1 macrophage rather than M2 macrophage. Since RGS12 OE not only promotes M1 macrophage polarization but also enhances ciliogenesis, we then determine if there are some relationships between M1 macrophages and cilia. Indeed, we found that M1 macrophages are positively related to the increased cilia number and length while M2 macrophages represent a negative correlation with ciliogenesis (Fig. 4f). Functionally, RGS12 OE promotes the TAM migration but does not affect the proliferation (Supplementary Fig. 4).

### Deletion of RGS12 in macrophages represses ciliogenesis and M1 macrophage polarization by promoting KIF2A expression

**Fig. 5:**
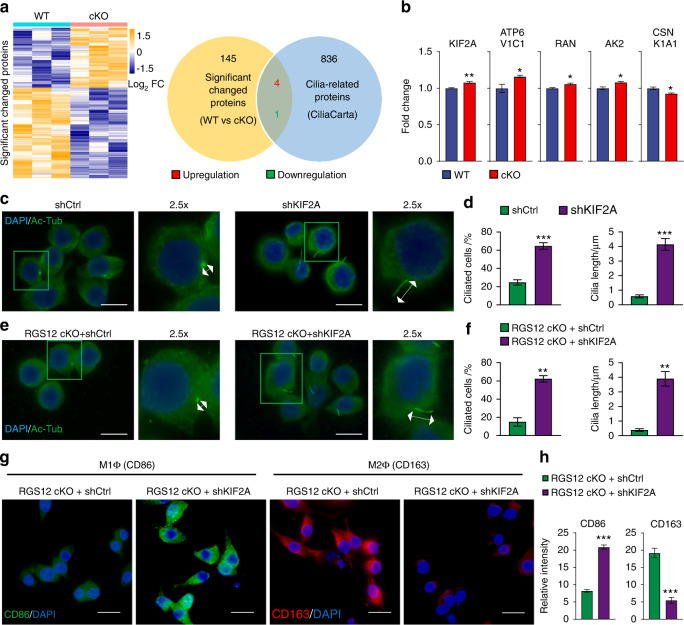
Deletion of RGS12 in macrophages represses ciliogenesis and M1 macrophage polarization by KIF2A. **a** Heatmap depicting the significantly changed proteins in RGS12 cKO macrophages. Venn diagram indicated the number of significantly changed proteins and ciliary proteins (CiliaCarta). **b** The significantly changed cilia proteins as indicated in **a**. *P < 0.05, **P < 0.01, *n* = 3. **c**, **d** The TAMs from WT mice were transfected with shCtrl or shKIF2A for 48 h. The ciliated cell number and cilia length were determined by immunofluorescence. Scale bar, 10 µm. ***P < 0.01, *n* = 5. **e**, **f** The TAMs from RGS12 cKO mice were transfected with shCtrl or shKIF2A for 48 h. The ciliated cell number and cilia length were determined by immunofluorescence. Data are expressed as mean ± SEM (**P < 0.01, *n* = 5). Scale bar, 10 µm. **g**, **h** Immunofluorescence to detect M1 (CD86) and M2 (CD163) macrophages in shCtrl and shKIF2A groups **g**. Scale bar, 20 μm. Statistical data showed the knockdown of KIF2A promotes M1 macrophages but inhibits M2 macrophage polarization **h**. ***P < 0.001, *n* = 5

To understand the interactions between RGS12 and cilia in macrophages, we implemented liquid chromatography-mass spectrometry (LC/MS) from the WT and RGS12 cKO BMMs (Fig. 5a). By analyzing the GO enrichment, we identified the top five biological processes (Supplementary Fig. 5), including cell cycle, protein transport, apoptotic process, innate immune response, and intracellular protein transport. Interestingly, these biological functions are closely related to the cilia protein functions.^32^ We also identified five proteins that involve in the regulation of cilia by conducting overlapping analysis with the CiliaCarta database (https://tbb.bio.uu.nl/john/syscilia/ciliacarta/) (Fig. 5a). Among these cilia proteins, kinesin family member 2 A (KIF2A) was the most increased protein in the RGS12 cKO macrophages (*P* < 0.01) (Fig. 5b). To determine the role of KIF2A in TAMs, we knocked down KIF2A by transfecting KIF2A shRNAs in primary TAMs isolated from oral cancer tissue of WT mice. The results showed that the knockdown of KIF2A in TAMs significantly enhanced the ciliated cell number and cilia length (Fig. 5c, d). We further knocked down KIF2A in RGS12 cKO TAMs and found that the downregulation of KIF2A still could increase the ciliated cell number and cilia length in RGS12 cKO TAMs (Fig. 5e, f). These results confirm that KIF2A acts downstream of RGS12 and RGS12 affects cilia formation and length by regulating KIF2A. To determine whether KIF2A is associated with macrophage polarization, we knocked down KIF2A by transfecting KIF2A shRNAs in RGS12 cKO TAMs and performing the immunofluorescence staining for detecting macrophage polarization marker genes. The results indicated that the knockdown of KIF2A in RGS12 cKO leads to increased CD86+ (M1 macrophage) cell numbers but decreased CD163+ (M2 macrophage) cells (Fig. 5g, h). These results demonstrated that RGS12 drives M1 polarization in TAMs by regulating the KIF2A-cilia signaling pathway.

**Fig. 6:**
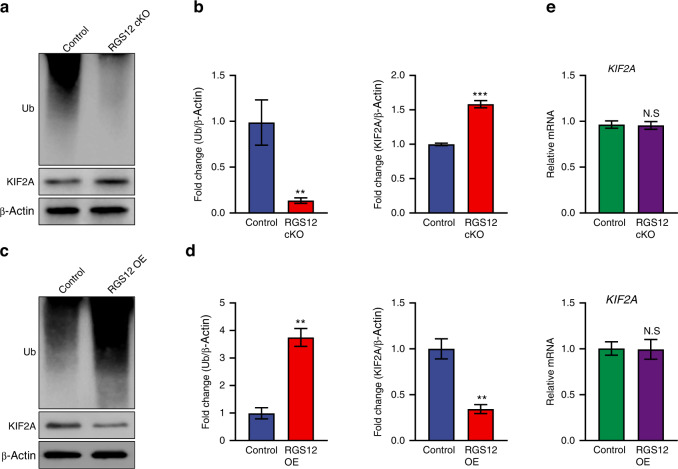
RGS12 promotes the ubiquitination and degradation of KIF2A in TAMs. **a**, **b** Western blot analysis of ubiquitin (Ub) and KIF2A expression in RGS12 cKO and control TAMs. Data are expressed as mean ± SEM (*n* = 3, **P < 0.01, ***P < 0.001). **c**, **d** The TAMs from WT mice were transfected with control (pCMV) and RGS12 OE (pCMV-RGS12) plasmids for 48 h. Western blot analysis of the ubiquitin (Ub) and KIF2A levels in TAMs. Data are expressed as mean ± SEM (*n* = 3, **P < 0.01). **e** Transcriptional levels of KIF2A in TAMs as described in **a**, **d**. Data are reported as means ± SEM (*NS* no significance, *n* = 5, *P* > 0.05)

Our previous study showed that RGS12 promotes ubiquitination.^26^ To test whether the increase of KIF2A in RGS12 cKO TAMs was caused by the change of ubiquitination, we tested the ubiquitination level. The result showed that the ubiquitination level was markedly decreased in RGS12 cKO TAMs but increased in RGS12 OE TAMs compared with the control group (Fig. 6a–d). Furthermore, the result of western blot confirmed that RGS12 regulation in KIF2A degradation evidenced as KIF2A protein level was increased in RGS12 cKO whereas decreased in RGS12 OE conditions (Fig. 6a–d), whereas the transcriptional expression levels of KIF2A did not change by the knockout or overexpression of RGS12 in TAMs (Fig. 6e), suggesting that RGS12 regulates KIF2A protein stability.

### RGS12 activates MYCBP2 by enhancing the p-Tyr in TAMs to decrease the KIF2A protein level

**Fig. 7:**
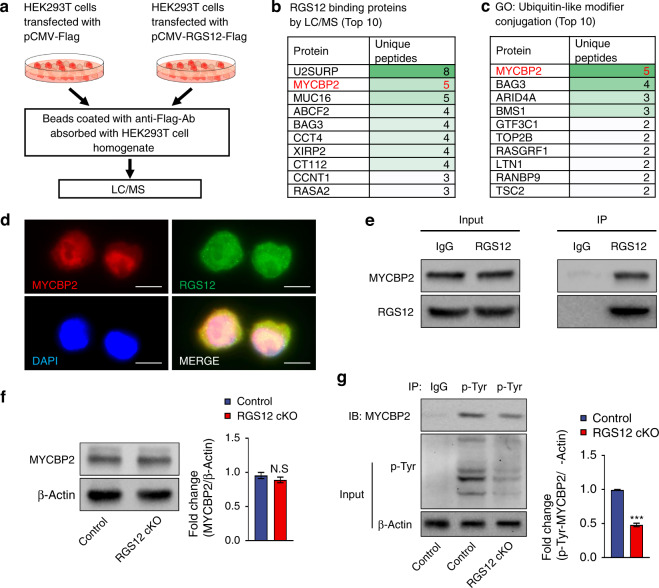
RGS12 activates MYCBP2 through p-Tyr in TAMs. **a** Identification of RGS12 interacting proteins. **b**, **c** The top ten unique proteins **b** and top ten ubiquitin-related proteins **c** were indicated in tables. **d** Immunofluorescence indicates that RGS12 and MYCBP2 colocalize in TAMs (Scale bar, 10 µm). **e** RGS12 associates with MYCBP2 in TAMs. The cell lysates were incubated with anti-RGS12 or immunoglobulin G (IgG) antibodies and the bound MYCBP2 was detected by immunoblotting. **f** TAMs from oral cancer tissues of Ctrl or RGS12 cKO mice were immunoblotted with an antibody against mouse MYCBP2. (*NS* no significance, *n* = 3, *P* > 0.05). **g** The TAM lysates from oral cancer tissues of Ctrl or RGS12 cKO mice were incubated with anti-p-Tyr or IgG antibodies, and bound protein was examined by western blot with the MYCBP2, p-Tyr, and β-Actin antibodies, as indicated. Note that RGS12 cKO decreases the phosphorylation (p-Tyr) of MYCBP2. ***P < 0.001, *n* = 3

To further understand the regulatory role of RGS12 on KIF2A, we identified the RGS12 interacting proteins by stably transfecting 293T cells with pCMV-RGS12-Flag or pCMV-Flag plasmids and implemented IP-LC/MS analysis (Fig. 7a). We identified the top ten unique proteins that bind with RGS12 (Fig. 7b). By implementing the gene enrichment analysis (Reactome), we found ubiquitin-related signaling such as E3 ligases target proteins and SUMOylation were the major signaling pathways in RGS12 binding proteins (Supplementary Fig. 6). Figure 7c showed the top ten ubiquitin-related proteins and the most significant protein in these proteins was MYC binding protein 2 (MYCBP2), suggesting that MYCBP2 may be the key molecule for RGS12-mediated ubiquitination. Consistently, RGS12 and MYCBP2 exhibited colocalization in TAMs by immunofluorescence staining (Fig. 7d). The immunoprecipitation assay further confirmed that RGS12 interacted with MYCBP2 in TAMs (Fig. 7e). Interestingly, we found that the loss of RGS12 did not affect the total protein levels of MYCBP2 (Fig. 7f) but decreased the phosphorylation (Tyr) of MYCBP2 in TAMs (Fig. 7g), demonstrating that RGS12 may activate MYCBP2 through phosphorylation.

### RGS12 promotes M1 macrophage polarization and ciliogenesis through MYCBP2 mediated KIF2A degradation

**Fig. 8:**
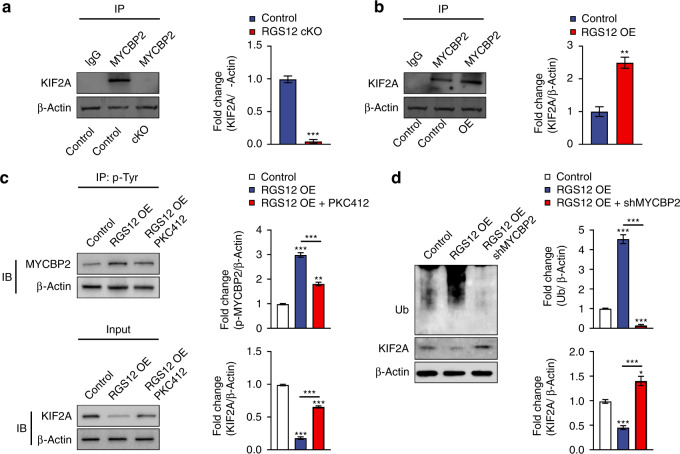
RGS12 promotes the degradation of KIF2A through MYCBP2. **a**, **b** TAMs were extracted from the RGS12 cKO mice or WT mice with oral cancer. The TAMs of WT mice were stably transfected with pCMV or pCMV-RGS12 plasmids. The cell lysates of TAMs were incubated with anti-MYCBP2 or IgG antibodies, and bound protein was detected by immunoblotting with the KIF2A antibody. **P < 0.01, ***P < 0.001, *n* = 3. **c** TAMs from oral cancer tissues of WT mice were stably transfected with pCMV (Ctrl) or pCMV-RGS12 (RGS12 OE) plasmids. Then, the TAMs were treated with PKC412 (1 μmol·L^−1^, tyrosine kinase inhibitor, Cayman) for 24 h. The cell lysates were incubated with anti-p-Tyr or IgG antibodies, and bound protein was examined by immunoblotting with the MYCBP2 antibody. Cell lysates were also immunoblotted with anti-KIF2A and anti-β-Actin. **P < 0.01, ***P < 0.001, *n* = 3. **d** TAMs from oral cancer tissues of WT mice were treated with pCMV (Ctrl) or pCMV-RGS12 (RGS12 OE) or pCMV-RGS12 + shMYCBP2 (RGS12 OE + shMYCBP2). Cell lysates were immunoblotted with anti-KIF2A, anti-Ub, and anti-β-Actin. Data are expressed as mean ± SEM (*n* = 3, *P < 0.05, ***P < 0.001)

**Fig. 9:**
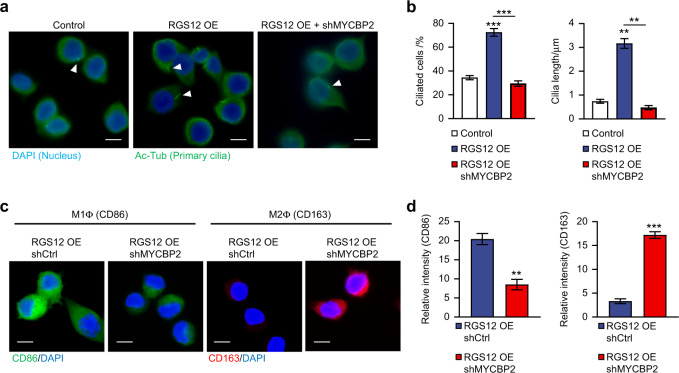
RGS12 promotes M1 macrophage polarization and ciliogenesis through MYCBP2. **a** TAMs from oral cancer tissues of WT mice were treated with pCMV (Ctrl) or pCMV-RGS12 (RGS12 OE) or pCMV-RGS12 + shMYCBP2 (RGS12 OE + shMYCBP2). Primary cilia were stained by immunofluorescent assay (Scale bar, 10 μm). **b** The ciliated cell numbers and cilia lengths of TAMs in (**a**) were analyzed by ImageJ software (NIH, US). Data are expressed as mean ± SEM (*n* = 5, **P < 0.01, ***P < 0.001). **c**, **d** TAMs were treated as described in **a**. The M1 macrophage (CD86) and M2 macrophage (CD163) were detected by immunofluorescence staining. Scale bar, 10 μm. **P < 0.01, ***P < 0.001, *n* = 5

To determine whether MYCBP2 is associated with RGS12 mediated decrease of KIF2A protein level, we performed the immunofluorescence of KIF2A and MYCBP2 in TAMs. The result showed that MYCBP2 and KIF2A were co-localized together in the cytoplasm and nucleus (Supplementary Fig. 7a). Immunoprecipitation results further demonstrated a strong association between MYCBP2 and KIF2A in TAMs (Supplementary Fig. 7b). Moreover, the loss of RGS12 inhibited the association whereas RGS12 OE enhanced the association between MYCBP2 and KIF2A in TAMs (Fig. 8a, b). To determine whether RGS12 enhances the degradation of KIF2A by controlling the phosphorylation of MYCBP2, we overexpressed RGS12 in TAMs and treated cells with PKC412 (a tyrosine kinase inhibitor) for 24 h. The results showed that RGS12 OE increased the p-MYCBP2 expression and promoted the degradation of KIF2A (Fig. 8c), which was inhibited by PKC412 (Fig. 8c). Moreover, we found that the inhibition of MYCBP2 suppressed the increased ubiquitination and decreased KIF2A caused by RGS12 OE (Fig. 8d). These results suggest that RGS12 mediates the degradation of KIF2A by controlling the phosphorylation of MYCBP2. To determine whether cilia changes in TAMs induced by RGS12 are regulated by MYCBP2, we transfected shMYCBP2 plasmids in the stably transfected RGS12 OE TAMs. The results indicated that RGS12 OE increased ciliated cell number and cilia length which were significantly repressed by the knockdown of MYCBP2 (Fig. 9a, b). We further examined if MYCBP2 regulates RGS12-mediated macrophage polarization. The results showed that the knockdown of MYCBP2 blocked RGS12 induced TAM polarization towards M1 macrophage (Fig. 9c, d).

## Discussion

TAMs are critical components in the tumor microenvironment that modulate the development of oral cancer.^33^ TAMs have the potential to eliminate cancer cells when properly re-educated.^34^ M1 TAMs exert an anti-tumor effect by producing pro-inflammatory cytokines.^35^ On the contrary, M2 TAMs hamper the pro-inflammatory cytokines and promote tumor progression.^35^ Thus, the promotion of macrophage polarization towards M1 macrophages has been considered a therapeutic strategy. Here, we found that loss of RGS12 in macrophages promotes oral cancer proliferation and invasion in the early stage. Moreover, the loss of RGS12 decreased M1 but increased M2 population, whereas the upregulation of RGS12 exhibited the opposite result. These findings suggest that RGS12 may be a key regulator for TAMs polarization towards the M1 phenotype, thereby creating an immunosuppressive environment for oral cancer.

It has been reported that primary cilia are located on monocytes from peripheral blood and bone marrow and are key organelles in modulating the Hh signaling.^18^ Recently, primary cilia were also found in dendritic cells from human skin and the number of ciliated cells was markedly upregulated in atopic dermatitis.^36^ Most surprisingly, primary cilia have essential functions in inflammatory signaling pathways,^37^ and the elongated cilia in fibroblasts were shown in IL1β induced inflammatory conditions.^38^ Consistent with these findings, we demonstrated that primary cilia are present in TAMs, and M1 TAMs had an increased length and number of cilia compared with M2 TAMs. Moreover, we also found for the first time that RGS12 can regulate the cilia formation and elongation in TAMs under inflammatory conditions.

RGS12 is a key player in inflammatory diseases due to its ability to associate with and/or activate key enzymes (e.g., COX2 and PTEN) as a scaffold protein.^25,39,40^ In this study, we found that RGS12 was associated with MYCBP2, an E3 ubiquitin-protein ligase, and enhanced the phosphorylation of MYCBP2 to degrade the ciliary protein KIF2A. This finding is supported by the finding that MYCBP2 locates in primary cilia, and ablation of MYCBP2 causes ciliopathy.^41^ However, how MYCBP2 regulates cilia remain unclear. Here, this study elucidated that RGS12 is required for MYCBP2-mediated KIF2A degradation and cilia disassembly in TAMs. In addition, RGS12 deficiency promotes TAMs polarization into M2 macrophages, which was blocked by the silence of MYCBP2. Supportively, Pierre et al demonstrated that the conditional knockout of MYCBP2 in macrophages drives the polarization of M2 macrophages in the inflamed tissue.^42^

**Fig. 10:**
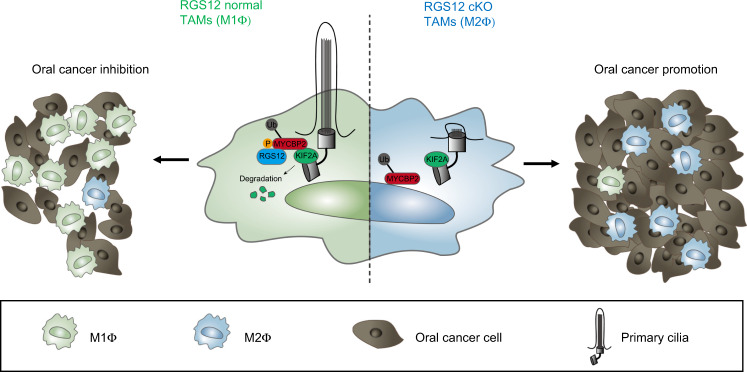
RGS12 promotes M1 polarization and ciliogenesis in TAMs. RGS12 associates with MYCBP2 and activates p-Tyr of MYCBP2 to degrade the cilia protein KIF2A in macrophages, which promotes the ciliogenesis, M1 polarization in TAMs and inhibits oral cancer. Loss of RGS12 leads to dysfunction of MYCBP2 and increases the KIF2A protein levels in macrophages, which inhibits ciliogenesis, activates M2 polarization in TAMs, and promotes oral cancer

In summary, this is the first study to demonstrate that RGS12 is required for TAMs M1 polarization and ciliogenesis via regulating MYCBP2 and KIF2A. Upregulation of RGS12 activates phosphorylation of MYCBP2 to enhance ciliogenesis by degrading KIF2A and promotes the M1 TAMs polarization to further eliminate oral cancer cells. However, downregulation of RGS12 in oral cancer inhibits the MYCBP2 activity, which further causes decreased ciliogenesis, increased M2 TAMs polarization, and promotes oral cancer growth (Fig. 10). Given that macrophage RGS12 is important for oral cancer development, targeting the RGS12/MYCBP2 pathway may be an attractive therapeutic avenue in this disease.

## Materials and methods

### Mouse models

The animal studies were implemented with the approval of the Institutional Animal Care and Use Committee of the University of Pennsylvania. All animals used in this study were a syngeneic, immunocompetent C57BL/6J mouse strain (The Jackson Laboratory, US). To generate the mice with macrophage-lineage conditional knockout of RGS12 (cKO), Rgs12^flox/flox(fl/fl)^ mice were mated with LysM-Cre mice.^40^

### Oral cancer models

Eight-week-old LysM-Cre control and RGS12 cKO littermates (*n* = 10) were generated to induce oral cancer models. The carcinogen 4-Nitroquinoline 1-oxide (4NQO, MilliporeSigma, US) stock solution was made at a concentration of 5 mg/mL. The mice were provided drinking water with a final concentration of 100 µg/mL 4NQO for 8 or 16 weeks and further changed to clean water for additional 4 weeks to observe the tongue tumorigenesis.^43^

### Histology

Mouse tongue tissues from each group were processed with an OCT embedding medium (Fisher Scientific, Hampton, NH, US) and kept at −80 °C. Frozen tongue tissues from LysM-Cre control and RGS12 cKO mice were sliced into 8-µm sections and performed a hematoxylin and eosin (H&E) stain to evaluate cancer pathology.

### Immunohistochemistry (IHC)

Human oral cancer tissue slides (OR601b) were obtained from the Biomax company (Derwood, US). Immunohistochemistry (IHC) of human oral cancer tissues was deparaffinized and hydrated and subjected to antigen retrieval. Then sections were incubated with RGS12 antibody (1:500, MilliporeSigma HPA054646, US), followed by a standardized avidin/biotin polymer technology using IHC Kit (Santa Cruz Biotechnologies sc-398545, US) according to the instruction.

### Immunofluorescence (IF)

Frozen tongue tissues or TAMs were fixed with 4% paraformaldehyde solution (MilliporeSigma, US) for 10 min at room temperature. Then, the sections were incubated with primary antibody [anti-acetylated α-Tubulin (Ac-Tub, 1:100, MilliporeSigma #T7451, US), anti-RGS12 (1:100, MilliporeSigma #GW21317, US), anti-KIF2A (1:200, Proteintech #13105-1-AP, US), or anti-MYCBP2 (1:100, MilliporeSigma #HPA058807, US)] and then incubated with the appropriate secondary antibody (1:500; Jackson ImmunoResearch Laboratory, US). A LAS-X (Leica) microscope was used to create maximum projections of z-stacks, and the fluorescence intensity, cilia length, and frequency were evaluated by ImageJ (NIH, US).^38^ All images were captured on a microscope with a comparable exposure time.

### Cell culture

Bone marrow macrophages (BMMs) from 8-week-old LysM-Cre control and RGS12 cKO mice were cultured in Dulbecco’s Modified Eagle Medium (DMEM) supplemented with 10% fetal bovine serum (FBS), 20 ng·mL^−1^ recombinant mouse M-CSF, and 100 U/mL Penicillin-Streptomycin (Thermo Fisher Scientific, US). Tumor-associated macrophages (TAMs), T cells, and B cells were isolated from oral cancer tissues by indirect magnetic labeling according to the kit instruction (Thermo Fisher Scientific #11203D, US). Briefly, TAMs were incubated in phosphate-buffered saline (PBS) consisting of different antibodies (anti-F4/80 #60027, anti-CD4 #60017, anti-CD19 #6006, STEMCELL Technologies, Cambridge, MA, US) for 30 min. Then, the cells were washed with PBS 2 times and mixed with magnetic beads. Labeled cells were separated by magnetic rack and harvested by the addition of DMEM. Serum starvation (12 h) was performed for the cilia studies.

### Plasmid construction and transfection

pCMV-RGS12 was created by cloning the mouse RGS12 open‐reading frame into p3×FLAG-Myc-CMV-26 (MilliporeSigma, US). shMYCBP2 (sc-155927) and shKif2A (sc-60884) were obtained from Santa Cruz (Dallas, TX, US). pCMV-KIF2A was obtained from Addgene (#52401, US). For cell transfection, TAMs from oral cancer tissues of WT mice were cultured in 6-well plates and transfected the indicated plasmids (3 µg) for 24 h using Lipofectamine 3000 reagent (Thermo Fisher Scientific, US). Subsequently, stably transfected cell clones were selected with appropriate antibiotics.

### WST-1 proliferation assay

TAMs were cultured in 96-well plates and the cell proliferation assay was performed under kit instructions (MilliporeSigma, US). Briefly, after culturing the cells for the indicated time periods, the culture medium was changed to 90 µL growth medium supplemented with 10 µL WST-1 solution and incubated at 37 °C for 1 h. The absorbance was read at 450 nm and 490 nm using a microplate absorbance reader (Bio-Rad Laboratories, Hercules, CA, US).

### Transwell assay

TAMs were harvested from LysM-Cre control and RGS12 cKO mice and cultured in the top chamber containing 8-µm-pore inserts in DMEM without FBS. DMEM supplemented with 10% FBS (750 μL) was added to each bottom chamber. The migrated TAMs were stained with 0.5% crystal violet (24 h) and counted by ImageJ software (NIH, US).

### Immunoprecipitation (IP)

HEK293T cells or TAMs were lysed in NP40 buffer containing a protein inhibitor cocktail and phenylmethylsulfonyl fluoride (MilliporeSigma, US). In brief, lysates containing equal contents of proteins (1 mg) were incubated at room temperature with anti-RGS12 (Santa Cruz Biotechnologies #sc-514173, US) or anti-MYCBP2 antibody (MilliporeSigma #MABN2397, US) for 1 h and next with protein A/G magnetic beads (Bio-Rad Laboratories #161-4833, US) overnight. After washing with TBST buffer 3 times, the precipitated proteins were quantified by western blot assay.

### Western blot (WB)

TAMs were lysed in radioimmunoprecipitation assay buffer with phenylmethylsulfonyl fluoride and protease inhibitor cocktail (MilliporeSigma, US). Proteins were separated by 10% SDS‐PAGE gel and transferred to 0.45 µm nitrocellulose (NC) membranes. The membranes were incubated with 5% skim milk for 1 h (room temperature) and next incubated with primary antibodies against RGS12 (1:1 000, MilliporeSigma #GW21317, US), KIF2A (1:1 000, Proteintech #13105-1-AP, US), MYCBP2 (1:1 000, Abcam #ab86078, UK), and β-Actin (1:5 000, Proteintech #66009-1-Ig, US) overnight (4 °C). The membranes were washed and incubated with secondary antibodies for 1 h. An enhanced chemiluminescent kit (Bio-Rad Laboratories, US) was used for imaging and quantitation by ImageJ software (NIH, US) after incubation with the secondary antibodies.

### Real-time PCR

RNA extraction from TAMs or oral cancer tissues was implemented by using TRIzol reagent (Thermo Fisher Scientific, US). Reverse transcription was performed by using PrimeScript RT Master Mix (Takara Bio, US). Real-time PCR was implemented by CFX96 Touch System (Bio-Rad Laboratories, US) and 2× SYBR Green qPCR kit (Bimake, US). Relative expression was calculated by the 2^−△△Ct^ method, with normalization to GAPDH expression. The primers were listed as follows: GAPDH (F): AGGTCGGTGTGAACGGATTTG, GAPDH (R): TGTAGACCATGTAGTTGAGGTCA; KIF2A (F): GCAGTGTTCCAGGAATCCAT, KIF2A (R): GCTTGCTTGCTTGCTCTTCT.

### Data acquisition and processing

The RNA sequencing data of human healthy and oral cancer were extracted from the Gene Expression Omnibus database (GSE37991 or GSE39400). A total of 20 samples of RNA sequencing data were included, including 10 samples of oral cancer patients and 10 samples of healthy subjects. The data were normalized through standard correction and logarithmic operation and combined into a data matrix. The data were analyzed by the R package and the DEGs with *P* < 0.05 and fold change ≥1.5 were considered statistically significant.

### Liquid chromatography–mass spectrometry (LC/MS)

The LC/MS experiment was introduced to compare the protein profiles of BMMs from LysM-Cre control and RGS12 cKO mice (8 weeks). A strict set of criteria was used for protein identification such as a low peptide and false discovery rate (FDR) of <0.05. Heat maps were created by the R package. The Database for Protein function (UniProtKB keywords), Annotation, Visualization, and Integrated Discovery (DAVID) was applied to analyze the Kyoto Encyclopedia of Genes and Genomes (KEGG) and Gene Ontology (GO) enrichment.

### Statistics

Statistical analyses were applied using GraphPad Prism 7. An unpaired 2-tailed t-test was used to compare differences between two groups. One-way ANOVA was applied to analyze changes among multiple groups. The correlation between the IF intensity counting was estimated by the coefficient of Person. The levels of Pearson correlations were described as no correlation (0), weakly (0.25), moderately (0.5), strongly (0.75), and perfectly positive correlation (1). Survival curves were evaluated with the Kaplan-Meier method from the Tumor Immune Estimation Resource (TIMER) platform. Data were presented as mean ± SEM, and *P* values less than 0.05 were considered significant.

## Supplementary information

Suppltmental Figures
