# Supplementary material for: RGS12 represses oral squamous cell carcinoma by driving M1 polarization of tumor-associated macrophages via controlling ciliary MYCBP2/KIF2A signaling
Source: Int J Oral Sci. 2023 Feb 16;15:11. doi: 10.1038/s41368-023-00216-5 (PMC9935888; doi:10.1038/s41368-023-00216-5)
Supplement: Supplementary file 1 — Suppltmental Figures [file 41368_2023_216_MOESM1_ESM.docx]

**
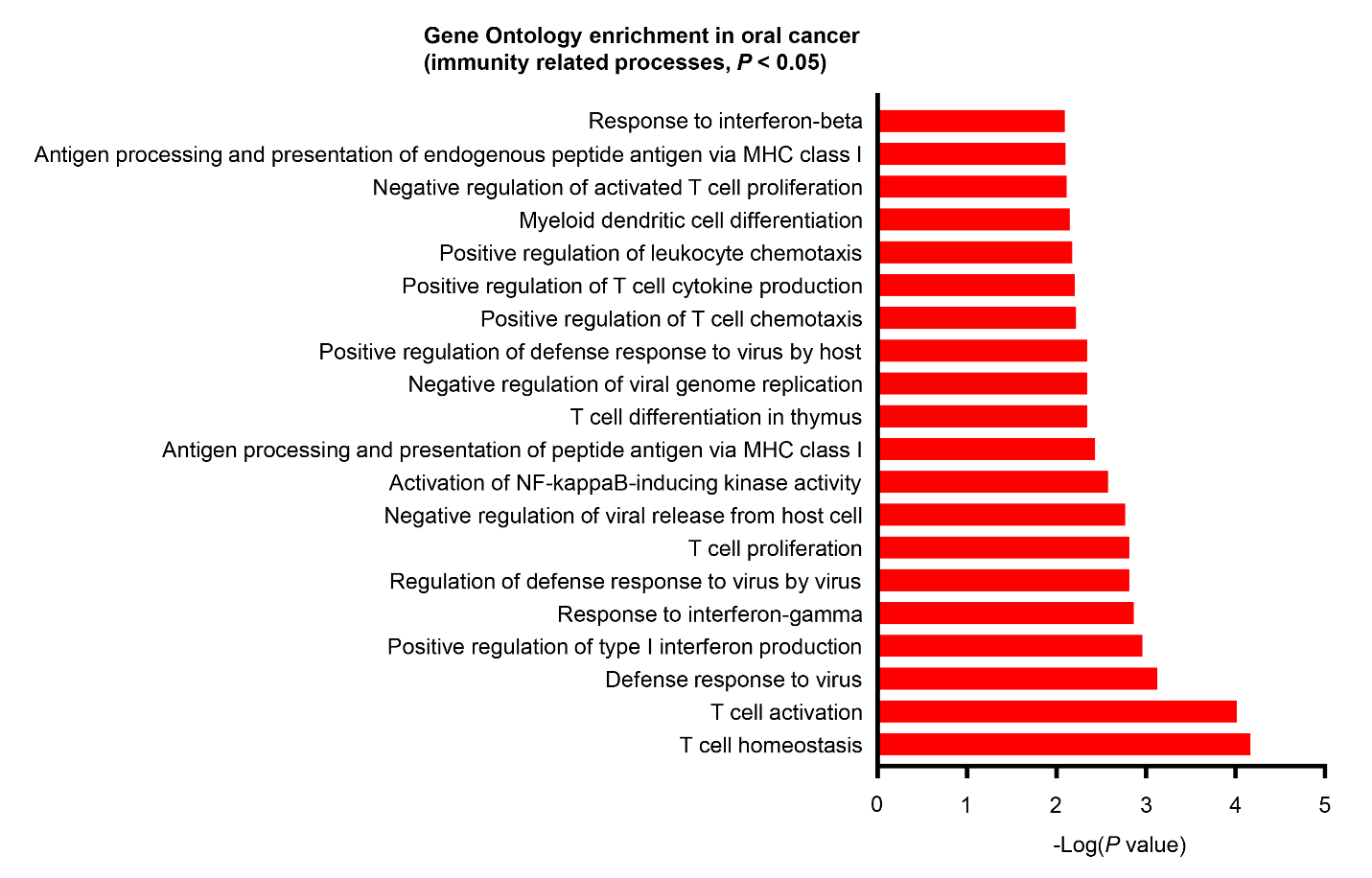
**

**Supplementary Fig.1 Gene Ontology analysis (immunity related processes) from human normal and oral cancer tissues**

The immunity related processes are enriched based on the differentially expressed genes between normal and oral cancer tissues. All GO categories with an adjusted enrichment *P* value of less than 0.05 and fold change greater than 1.5 are included in the figure (*n* = 10).


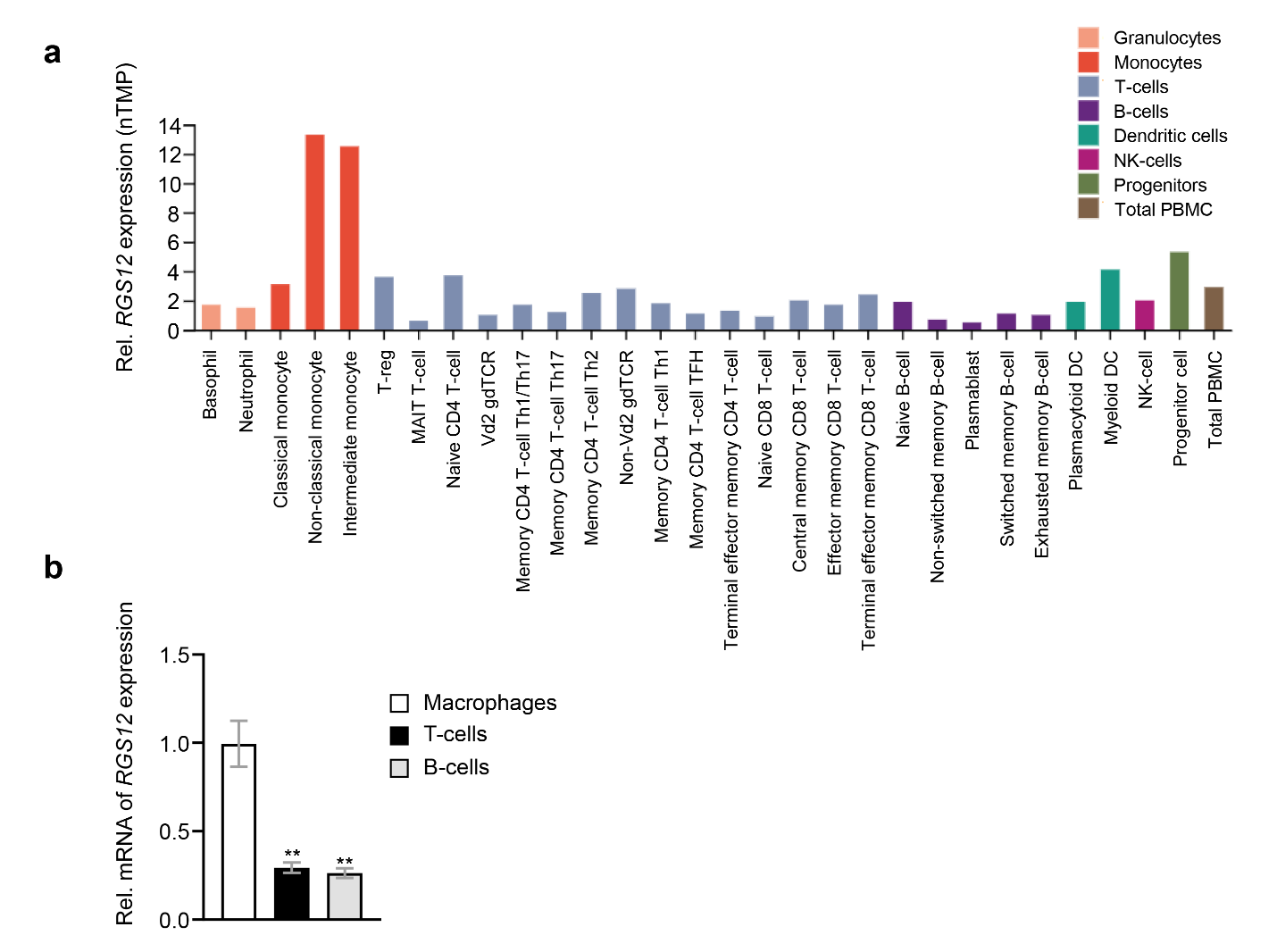


**Supplementary Fig. 2 RGS12 is more highly expressed in monocytes than in other immune cells**

(**a**) The relative mRNA expression levels of *RGS12* were analyzed in human peripheral blood mononuclear cells (Monaco scaled dataset) through the Protein Atlas (https://www.proteinatlas.org/ENSG00000159788-RGS12/blood). The results showed the *RGS12* mRNA levels in different cell lineages (pTPM, protein-coding transcripts per million). Note that *RGS12* was more highly expressed in monocytes than in other cell types.

(**b**) Macrophages, T cells, and B cells were isolated from the oral cancer tissues with magnetic beads conjugated with antibodies against surface markers (macrophages, F4/80; T cells, CD4; B cells, CD19). The relative mRNA expression levels of *RGS12* were determined by real-time PCR. Data are presented as the mean ± SEM. ***P* < 0.01, *n* = 3.


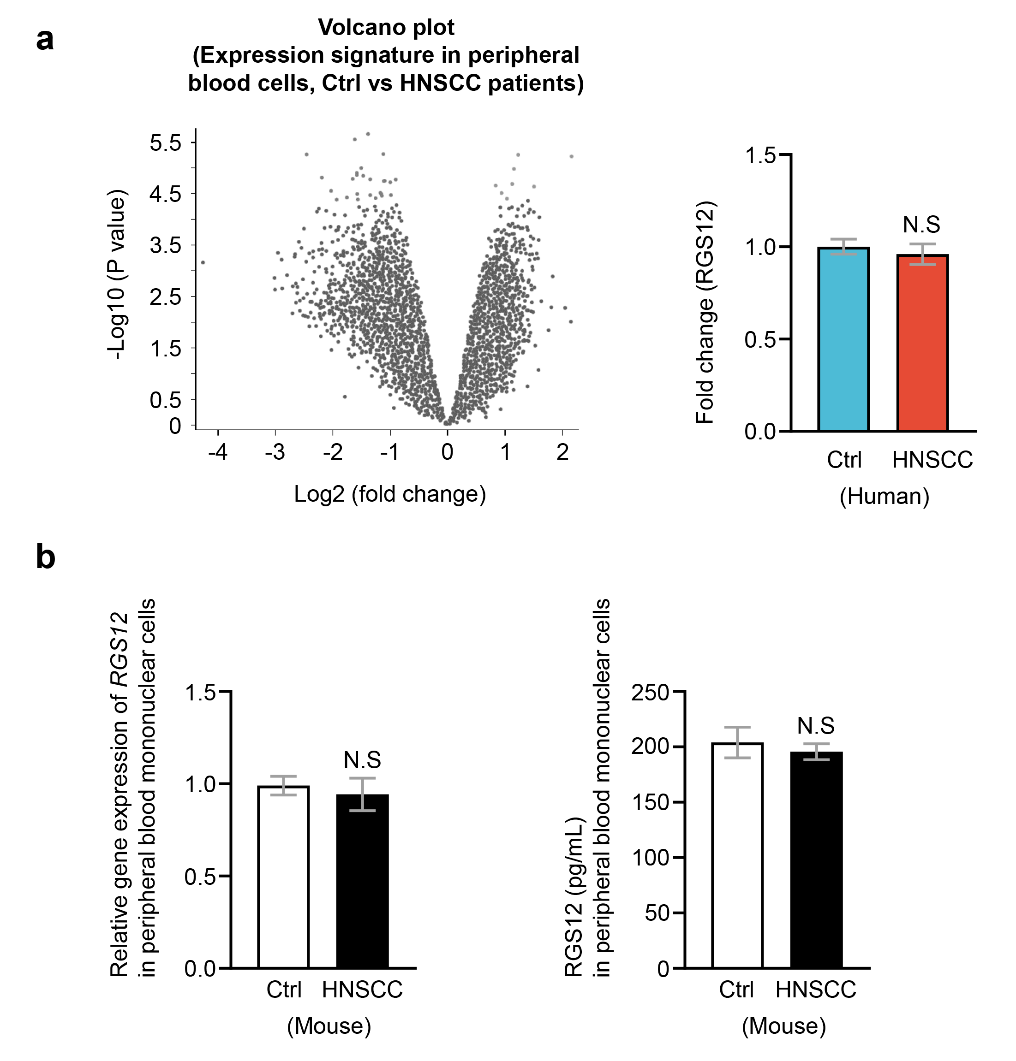


**Supplementary Fig. 3 RGS12 in peripheral blood cells showed no difference between the HNSCC patients and healthy control people**

(**a**) Volcano plot of DEGs in peripheral blood cells from patients with head and neck squamous cell carcinoma (HNSCC) compared with healthy control people (Database: GSE39400, *n* = 10). The x-axis represented the log2 fold change and the y-axis represented the log10 (P-value). The right panel showed the relative RGS12 gene expression in peripheral blood cells. Note that there was no significance (N.S, *P* > 0.05) between the HNSCC patients and healthy control people.

(**b**) The relative mRNA expression and protein expression of RGS12 in peripheral blood mononuclear cells from the healthy control mice and HNSCC mice. Note that there was no significance between the healthy control mice and HNSCC mice (N.S, *P* > 0.05, *n* = 5).


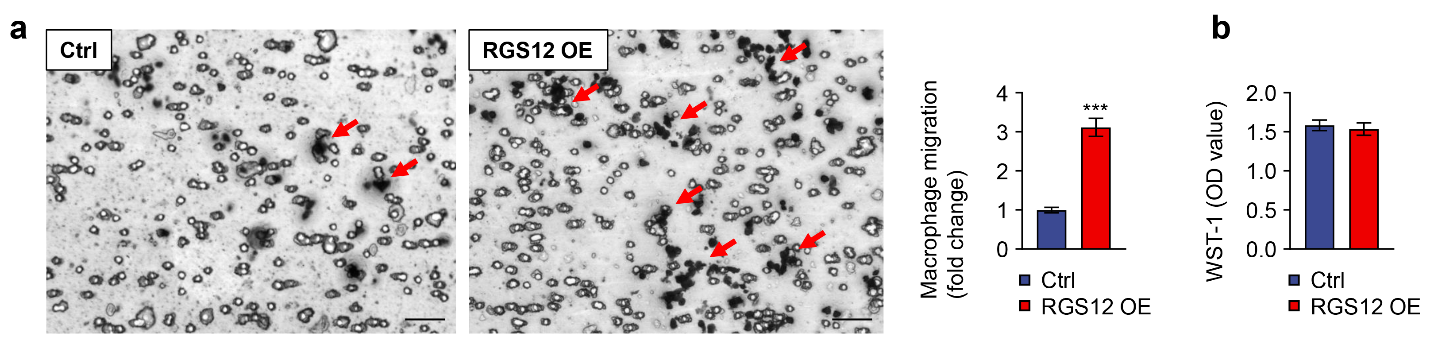


**Supplementary Fig. 4** **RGS12 promotes the TAMs migration but not proliferation**

(**a**) Representative images from Transwell assays in TAMs transfected with pCMV-RGS12 (RGS12 OE) or control vector (Ctrl) for 24 h. Migrated TAMs (red arrows) were detected at the bottom of the Transwell insert by 0.5% crystal violet staining. The numbers of TAMs were analyzed by ImageJ software. Scale bar, 50 μm. ****P* < 0.001, *n* = 5.

(**b**) Cell proliferation for TAMs was measured by the WST-1 assay. *P* > 0.05, *n* = 5.


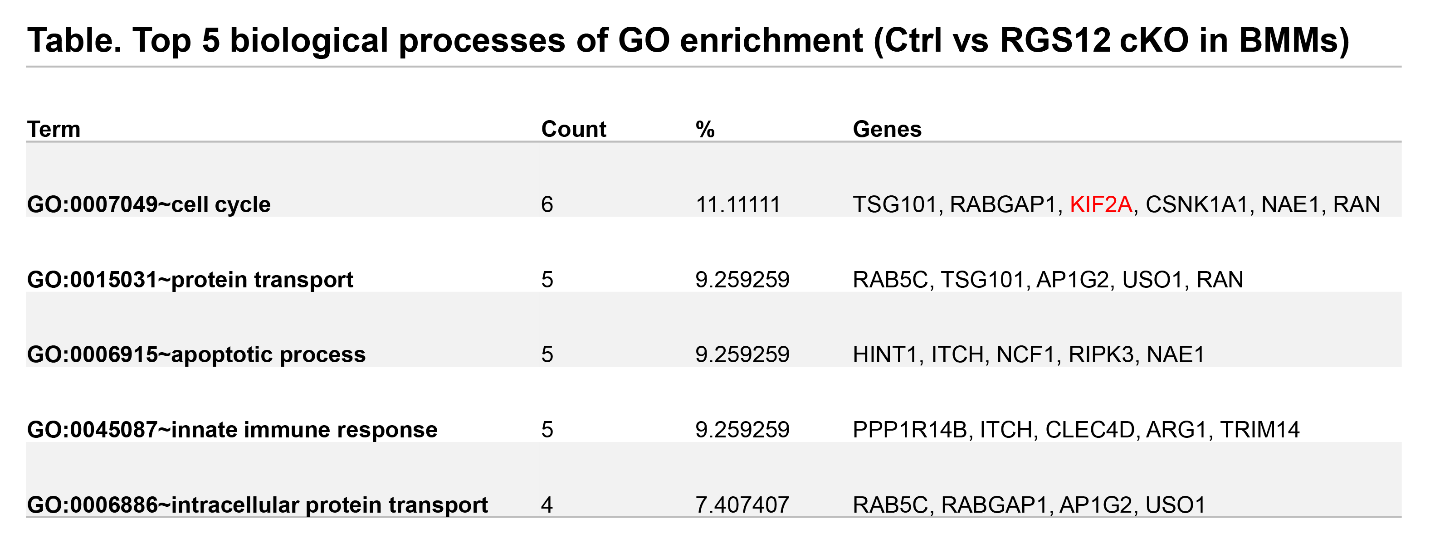


**Supplementary Fig. 5 Top 5 biological processes of GO enrichment in WT and RGS12 cKO BMMs**

Gene Ontology (GO) annotation was used to perform functional enrichment analysis using the DAVID tools. The number of genes refers to the number of differentially expressed genes described by that annotation and is also defined as a percentage of the total genes described for that term. The table represents the top five terms within the biological processes of Gene Ontology.

**Table.** **Top 5 signaling pathways of RGS12 binding proteins**

| Pathway identifier | Pathway name | #Entities found | #Reactions  found |
| --- | --- | --- | --- |
| R-HSA-72203 | Processing of Capped Intron-Containing Pre-mRNA | 9 | 24 |
| R-HSA-3108232 | E3 ligases target proteins | 7 | 25 |
| R-HSA-72163 | mRNA Splicing - Major Pathway | 7 | 8 |
| R-HSA-2990846 | SUMOylation | 7 | 25 |
| R-HSA-3000178 | ECM proteoglycans | 6 | 6 |

**Supplementary Fig. 6 Reactome analysis showed the top 5 signaling pathways (RGS12 binding proteins)**

Signaling pathways were analyzed by using the Reactome tools. The entities found refers to the number of differentially expressed proteins. The table represents the top five signaling pathways within the Reactome analysis. Note that RGS12 binding proteins are mainly involved in the E3 ligases target proteins and SUMOylation.


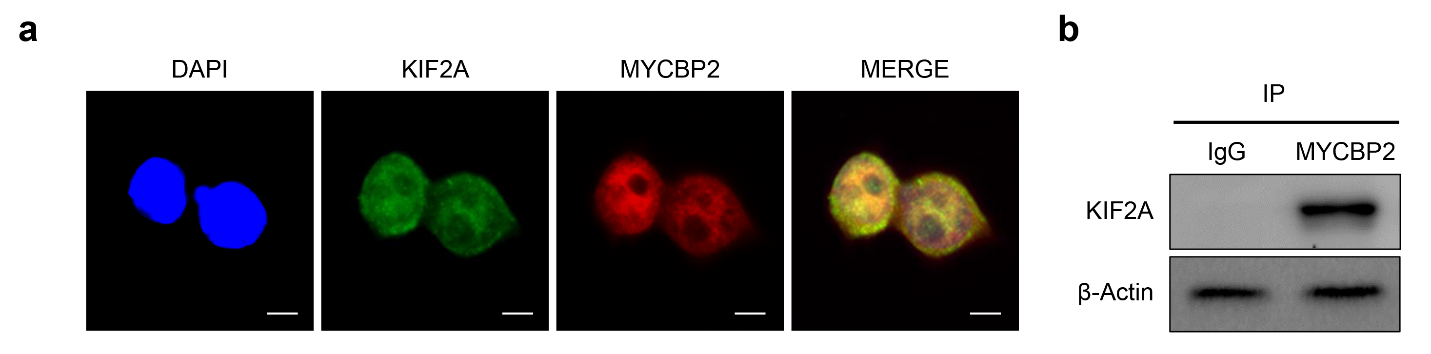


**Supplementary Fig. 7 MYCBP2 associates with KIF2A in TAMs**

(**a**) Immunofluorescence showed that MYCBP2 colocalized with KIF2A in TAMs from WT mice with oral cancer. Scale bar, 5 µm.

(**b**) MYCBP2 associates with KIF2A in TAMs. Antibody to MYCBP2 or a control IgG was incubated with TAMs extracts. Immunoprecipitated proteins were probed for KIF2A.
